# Supplementary material for: Chromothripsis during telomere crisis is independent of NHEJ, and consistent with a replicative origin
Source: Genome Res. 2019 May;29(5):737–49. doi: 10.1101/gr.240705.118 (PMC6499312; doi:10.1101/gr.240705.118)
Supplement: Supplemental Material [file supp_gr.240705.118_Supplemental_file_1.zip › contigs/annotated_contigs/DB83/contig.3.DB83_length_1028_mean_cov_5.25486381323.docx]

**DB83_length_1028_mean_cov_5.25486381323**

```
CTCTTAACCATTTCCCTCCTTCCCACACCCCACTCTTCACACATACATGTACATCCCTACCTGAGCTAGAGCCCTGCTTCCATCCACTT
 >chr14:72732411-72732724 + E=3e-161 p=3e-03
TCCTGGCCTTCCCCAAGCTGCCTGTGCAAAGCCTCCCTTTTCTGCTCCCTCAGCCTCCGGTTTCTTCCTTTTTCTATACCTTTCCCCAA

CAATTTCAGCCTTTATTAACCTTTTCCTTCTCTAACTATCAGTCCTATCGCTTTTTTTTTGGGGGGGGGGAGGCAGGTGGGAGGTGTAC

AGCTCCTCATACTTGCTGTTCTGTTTATGTATGCTCTATA|GCAA|GCTCTACCTCTAGCAACGTCACTACTAATGGGATGAGATCAAG
                                         >chr14:72731803-72732269 - E=3e-262
CAGCCTGTCACTTAAAAACAGACAAACACGACAACCCTACAGCCTGTCTGTGGTTCCCAAGAGGTCTTCTCCACCCTTGAGGGCACTGA

CCAGTACCTGGCAGCACATGCTCCATTTAACCCCCAGGCCTGCGTCTGAGCCAGGTGGCAGGATTTCCCATGAAGCAAGGAGAGAGACA

CAGGGTCAAGGGGCTTGGACCCAAAATGCCGCATTGGCCACAGAGTTGCCTGGATTGCTGGTTTTCTGATCCCTCATGTATGCAAGGAT

GCCCAGCACCGTGTCACCATGCCCACCACACCCCACTAATTTAACACCACTAATTCAATCAAATCCAAAGTGTTTTTCAAATTAGGACA

TTTCACATACACTTAGCCAACCTGAAAATCTTCATACTGAGTATTTCCACCATCCTGAATT|TT|TCCCTATCATCACTCATGATTTTG
                                                              >chr14:72732761-72733018
TGGATCGGGAATTCCAGCCGGACTCAGCTGGGAAATTCTTTTGCTCCTCGCTGTATTGAAGGGGGTCGCTGGATACTTTCACTGGGCAG
+ E=1e-142
ATGGGTGAACTGGAGGTGATTAAGATGGCTTCACATGCCTGGCGCCTTGGTGGGATGGCTGGAAGGCATGTTCAGCTGGGTGTGTCCAC

TGGGGCTTCCACACATGGTGGTCTCAAAGTAGTCAGACCTCTTTCAGGTAGCT
```
